# Operative Case Volumes and Variation for General Surgery Training in East, Central, and Southern Africa

**DOI:** 10.1007/s00268-023-07164-5

**Published:** 2023-09-11

**Authors:** Michael M. Mwachiro, Yves Yankunze, Niraj Bachheta, Emma Scroope, Deirdre Mangaoang, Abebe Bekele, Russell E. White, Robert K. Parker

**Affiliations:** 1 Department of Surgery, Tenwek Hospital, PO Box 39, Bomet, 20400 Kenya; 2 College of Surgeons of East, Central, and Southern Africa, Arusha, Tanzania; 3 Institute of Global Surgery, Royal College of Surgeons in Ireland, Dublin, Ireland; 4 Department of Surgery, Alpert Medical School of Brown University, Providence, RI USA; 5 University of Global Health Equity, Kigali, Rwanda

## Abstract

**Background:**

Operative experience is a necessary part of surgical training. The College of Surgeons of East, Central, and Southern Africa (COSECSA), which oversees general surgery training programs in the region, has implemented guidelines for the minimum necessary case volumes upon completion of two (Membership) and five (Fellowship) years of surgical training. We aimed to review trainee experience to determine whether guidelines are being met and examine the variation of cases between countries.

**Methods:**

Operative procedures were categorized from a cohort of COSECSA general surgery trainees and compared to the guideline minimum case volumes for Membership and Fellowship levels. The primary and secondary outcomes were total observed case volumes and cases within defined categories. Variations by country and development indices were explored.

**Results:**

One hundred ninety-four trainees performed 69,283 unique procedures related to general surgery training. The review included 70 accredited hospitals and sixteen countries within Africa. Eighty percent of MCS trainees met the guideline minimum of 200 overall cases; however, numerous trainees did not meet the guideline minimum for each procedure. All FCS trainees met the volume target for total cases and orthopedics; however, many did not meet the guideline minimums for other categories, especially breast, head and neck, urology, and vascular surgery. The operative experience of trainees varied significantly by location and national income level.

**Conclusions:**

Surgical trainees in East, Central, and Southern Africa have diverse operative training experience. Most trainees fulfill the overall case volume requirements; however, further exploration of how to meet the demands of specific categories and procedures is necessary.

## Introduction

There is a tremendous disparity in the lack of access to surgical care for billions of people worldwide [1]. This gap is especially evident in East, Central, and Southern Africa. Robust surgical training is vital to creating competent surgeons capable of improving access to quality care [2]. The College of Surgeons of East, Central, and Southern Africa (COSECSA) was established to facilitate the training of surgeons and is the largest surgical training institution in Africa [3]. The five-year general surgery training program, with a set curriculum, comprises an initial Membership level (2 years) followed by a Fellowship level (3 years) [4].

Various factors contribute to surgical training, but operative case experience is understood to be necessary. Trainee operative case volumes are associated with improved scores on technical skills and work-based assessments [5]. Trainees obtain better objective skill performance scores with increased operative encounters [6]. Case volumes have been examined in various general surgery training paradigms [7], with an estimated average of 1366 operative training cases [8]. Most studies are from high-resource settings, but there are reports within low- and middle-income countries [9–14]. A recent study examining multiple training programs in countries in Africa described the case volumes of trainees with diverse experience and a high volume of cases [15]. Previously, a group of surgeons and educators proposed a list of desired minimums for general surgery training in the region [16]. In 2019, a panel from COSECSA proposed guideline minimums for the number of operations for each trainee. Yet, knowledge about the volume and types of procedures available to meet guideline minimums within COSECSA training is lacking.

To provide a benchmark for East, Central, and Southern Africa, the aim of this study was to contribute to the regional understanding of surgical training by describing operative cases performed by trainees in multiple countries in East, Central, and Southern Africa, comparing these experiences to other published recommendations, and reviewing their fulfillment of the guidelines for minimum volumes.

## Materials and methods

We reviewed the operative case logs of a cohort of general surgery trainees. The study received IRB approval from the College of Surgeons of East, Central, and Southern Africa. The COSECSA eLogbook was implemented in 2015, and all COSECSA trainees are expected to record operative cases in the database. COSECSA trainees within the Pan-African Academy of Christian Surgeons training programs are exempt because they have an ongoing case log system, and therefore some of these trainees are not included in this review [15, 17]. We reviewed operative case logs from trainees enrolled in the FCS (Fellows of COSECSA) General Surgery program or trainees enrolled in the MCS (Members of COSECSA) program from 1st January 2015 to 31st December 2020. In August 2021, when we queried the system, there were 270,078 operations recorded by 769 trainees, as having been performed between those dates. Individuals who had completed the years of training, two years for MCS and three years for FCS, were included in this review.

Operative details contained the date of operation, description of operation, and location of operation. Operations performed while trainees were not enrolled in COSECSA training were excluded. However, procedures performed by a COSECSA trainee at COSECSA unaccredited hospitals were included to represent the experience of the trainees. We categorized each operation into defined categories. The COSECSA Panel Head had previously determined these categories for General Surgery, and in 2019 a working group established guideline minimum numbers for the categories. Individual operations from the case log entries were reviewed by an author (RP) and classified into the previously defined categories that are mapped to specific procedures. We validated the procedural categorization by examining a subset of random procedures selected using the Microsoft Excel random number generator function. A total of 1530 operations were then reviewed by two other surgeons (MM, YY). We then assessed agreement with Cohen’s kappa statistic. To better understand the differences from geography and development indices, we examined case logs from each country and compared the case volumes in low-income and lower-middle-income countries, as defined by the World Bank [18].

The primary outcome was the overall operative case volume. The secondary outcome was the total for each defined category. We examined the number of cases recorded by FCS trainees with the requirements from the Accreditation Council for Graduate Medical Education (ACGME) and with survey-based recommendations from regional surgeon educators [16, 19]. We used descriptive statistics and made comparisons using nonparametric tests because normality could not be assumed. We considered a p-value of 0.05 to be statistically significant. We organized data in Microsoft Excel (Redmond, WA, USA) and performed the analysis with Stata version 16.0 (College Station, TX, USA).

## Results

**Fig. 1:**
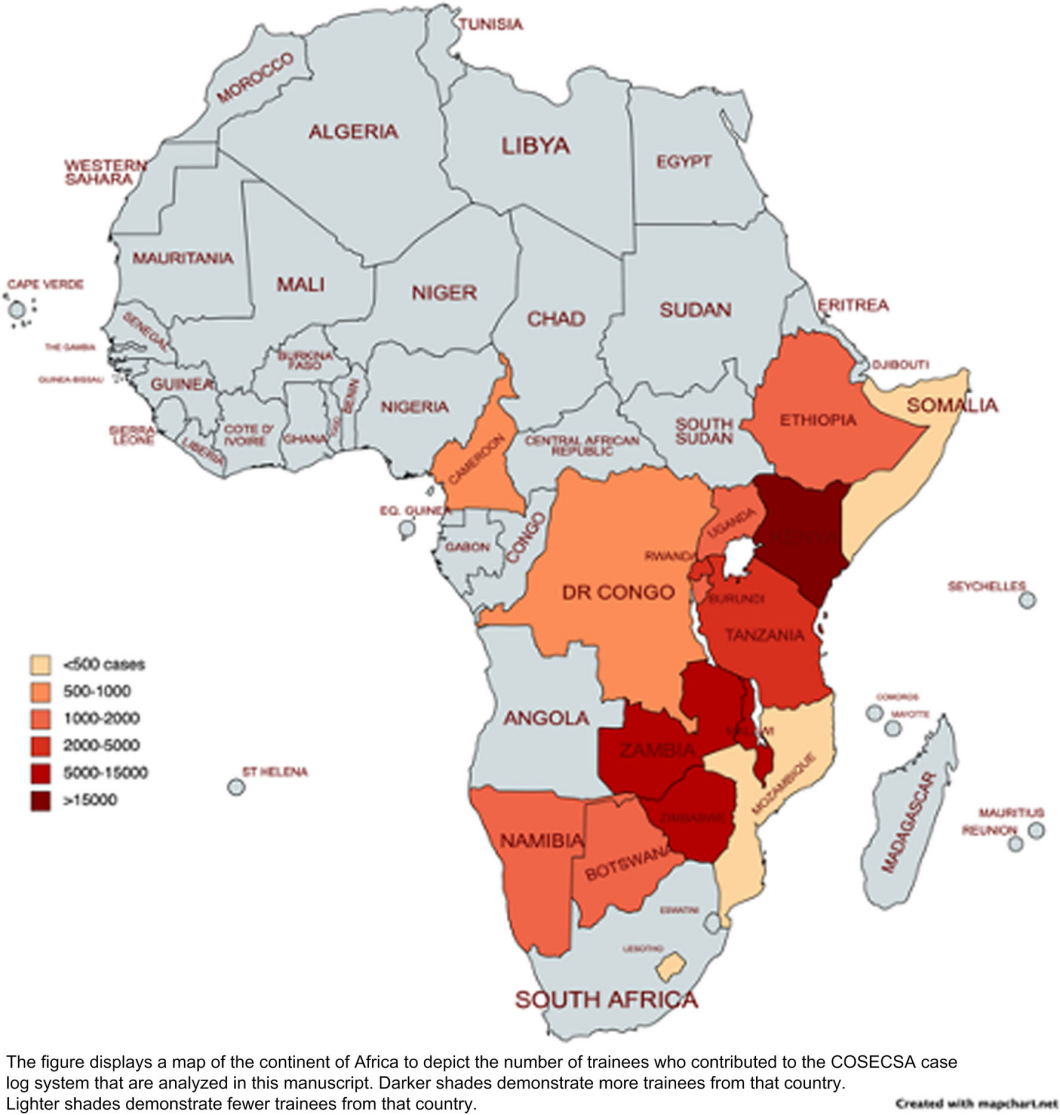
Map of countries with COSECSA training centers contributing to the database

**Fig. 2:**
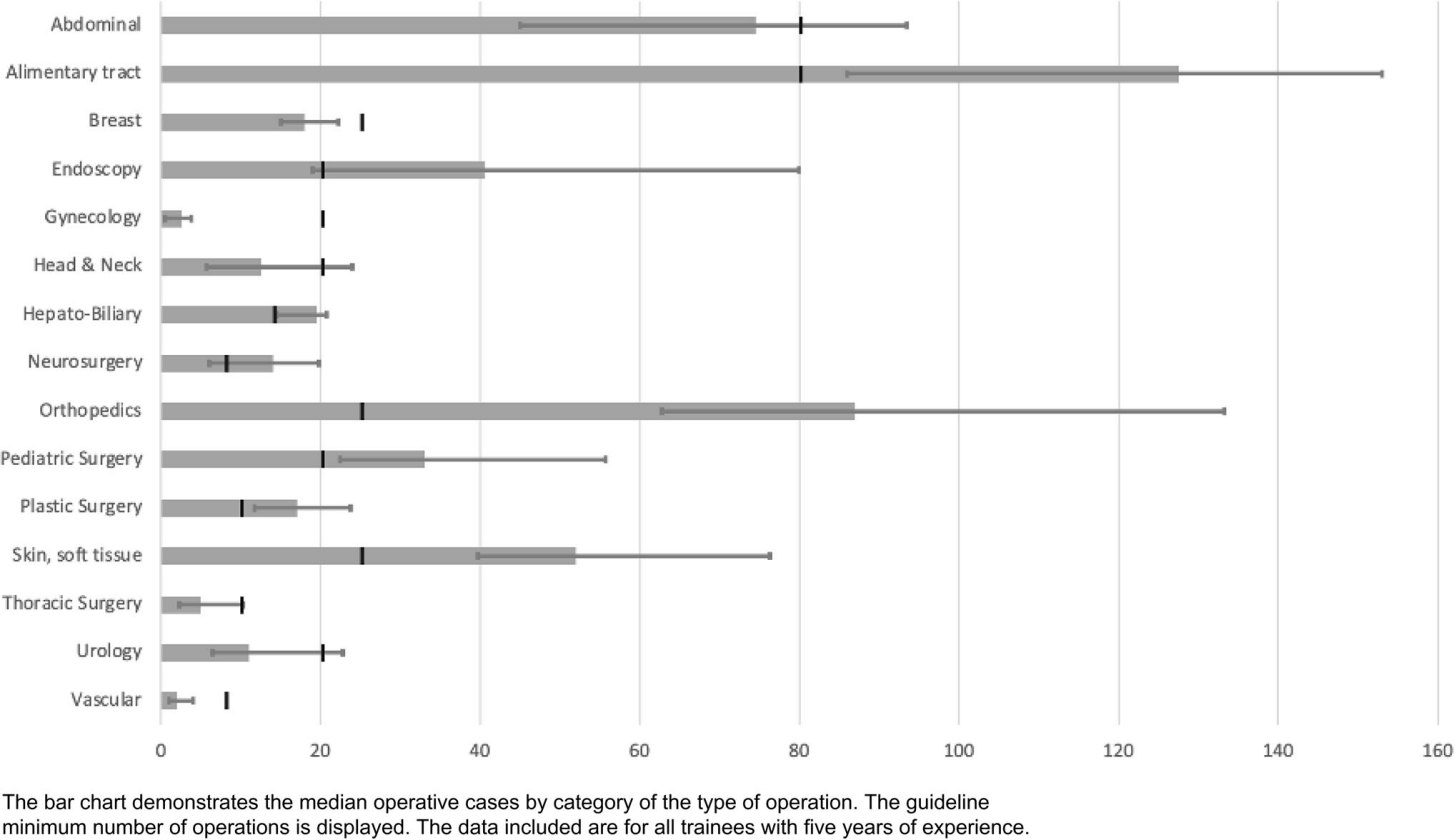
Bar chart demonstrating the median operative cases by category with the guideline minimum number for trainees with five years of experience

Of the 194 trainees included, there were 69,283 unique procedures to review and classify. The location of the operation by country is displayed in Fig. 1. There were 53,368 operations logged by 177 MCS level trainees and 15,895 operations logged by 31 FCS level trainees (14 trainees had both MCS and FCS level cases–5 years of complete training). There was substantial agreement within the validation subset with a Cohen’s kappa of 0.72. For MCS trainees, the distribution of cases by procedure and comparison to the defined guideline minimum volumes is displayed in Table 1. Eighty percent of trainees met the guideline minimum for 200 overall cases; however, numerous trainees did not meet the guideline minimum for each procedure. Table 2 demonstrates the FCS operations by category and guideline minimums. The median case volumes of COSECSA trainees would be adequate for ACGME requirements for plastic surgery, pediatric surgery, and skin and soft tissue categories. COSECSA trainees would not meet the desired minimums proposed by a prior survey of surgeons and educators in the region [16]. Figure 2 also displays the case volumes by category. All FCS trainees met the volume target for overall cases and orthopedics; however, numerous trainees did not meet the guideline minimums for the other categories.

**Table 1:** Operative case volumes by category for membership-level trainees (first two years)

Category	COSECSA guideline minimum	Median	25% quartile	75% quartile	Percent met guideline minimum
Abdomen-closure of intestinal perforation/bleeding ulcer	2	2	0	4	54.8
Abdomen-creation of intestinal stoma	3	5	2	10	67.8
Abdomen-intestinal anastomosis	5	10	5	16	80.2
Abdomen-laparotomy (including trauma)	10	16	10	23	75.7
Amputation (all types)	5	9	6	15	81.9
Anal and Perineal Surgery	5	7	3	11	59.9
Anesthesia-intubation, surgical airway	5	0	0	2	18.6
Anesthesia-regional including spinal	5	0	0	0	4.0
Appendicectomy	5	8	4	13	72.9
Biopsy-Fine Needle Aspiration or Cutting biopsy needle	5	3	1	7	42.4
Biopsy or excision of subcutaneous or deep mass (including lymph node, excluding breast)	10	11	6	19	58.8
Breast Surgery (Biopsy, lumpectomy, mastectomy)	5	6	3	9	56.5
Central venous access	2	0	0	2	28.2
Chest tube	5	2	0	6	35.0
Closure of complex wounds	5	22	11	35	94.4
Endoscopy (all types of GI)	5	1	0	6	29.9
Hernia-abdominal wall	3	5	2	8	71.8
Hernia-inguinal (all types)	5	12	7	21	84.7
Incision and drainage of abscess	10	12	6	22	61.6
Non-operative trauma care	3	0	0	0	0.0
Ob-gyn-Adnexal surgery	3	0	0	1	10.2
Ob-gyn-D&C	4	0	0	0	0.6
Ob-gyn-Caesarean section	5	0	0	1	15.3
Orthopedics-closed reduction of fractures & dislocations	5	34	15	61	92.1
Orthopedics-fasciotomies, carpal tunnel	2	0	0	1	23.7
Orthopedics-open fractures and/or external fixator	5	3	1	5	29.9
Other	2	38	23	72	98.9
Skin grafts	8	7	4	11	48.6
Urology-Circumcision	5	2	0	6	33.9
Urology-Prostatectomy	2	1	0	4	44.6
Urology-Suprapubic cystostomy	5	1	0	4	17.5
Total	200	248	204	360	80.2

**Table 2:** Operative case volumes by category for trainees at the fellowship level (all five years)

Category	ACGME Minimum	Regional Desired Minimum	COSECSA guideline minimum	Median	25% quartile	75% quartile	Percent met guideline minimum
Abdominal	250 (includes 90 below)	180	80	74.5	45	93.5	50.0
Hepato-Biliary	90	–	14	19.5	14.5	20.75	71.4
Alimentary tract	180	190	80	127.5	86	153	78.6
Breast	40	35	25	18	15	22.25	21.4
Endoscopy	85	60	20	40.5	19	80	71.4
Gynecology	0	50	20	2.5	0.5	3.75	7.1
Head & neck	25	40	20	12.5	5.75	24	35.7
Neurosurgery	0	–	8	14	6	19.75	50.0
Orthopedics	0	120	25	87	62.75	133.25	100.0
Other	–	–		281.5	213.25	320.5	-
Pediatric surgery	20	40	20	33	22.5	55.75	85.7
Plastic surgery	10	40	10	17	11.75	23.75	92.9
Skin, soft tissue	25	60	25	52	39.75	76.25	85.7
Thoracic surgery	20	15	10	5	2.25	10.25	28.6
Urology	0	60	20	11	6.5	22.75	42.9
Vascular	50	40	8	2	1	4	21.4
Total	850	1000	385	847.5	721.5	997.75	100

**Fig. 3:**
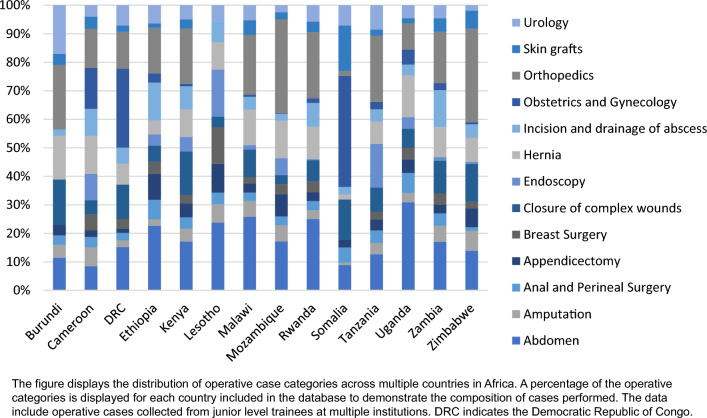
Distribution of junior surgical trainee operative case categories across multiple countries in Africa

**Fig. 4:**
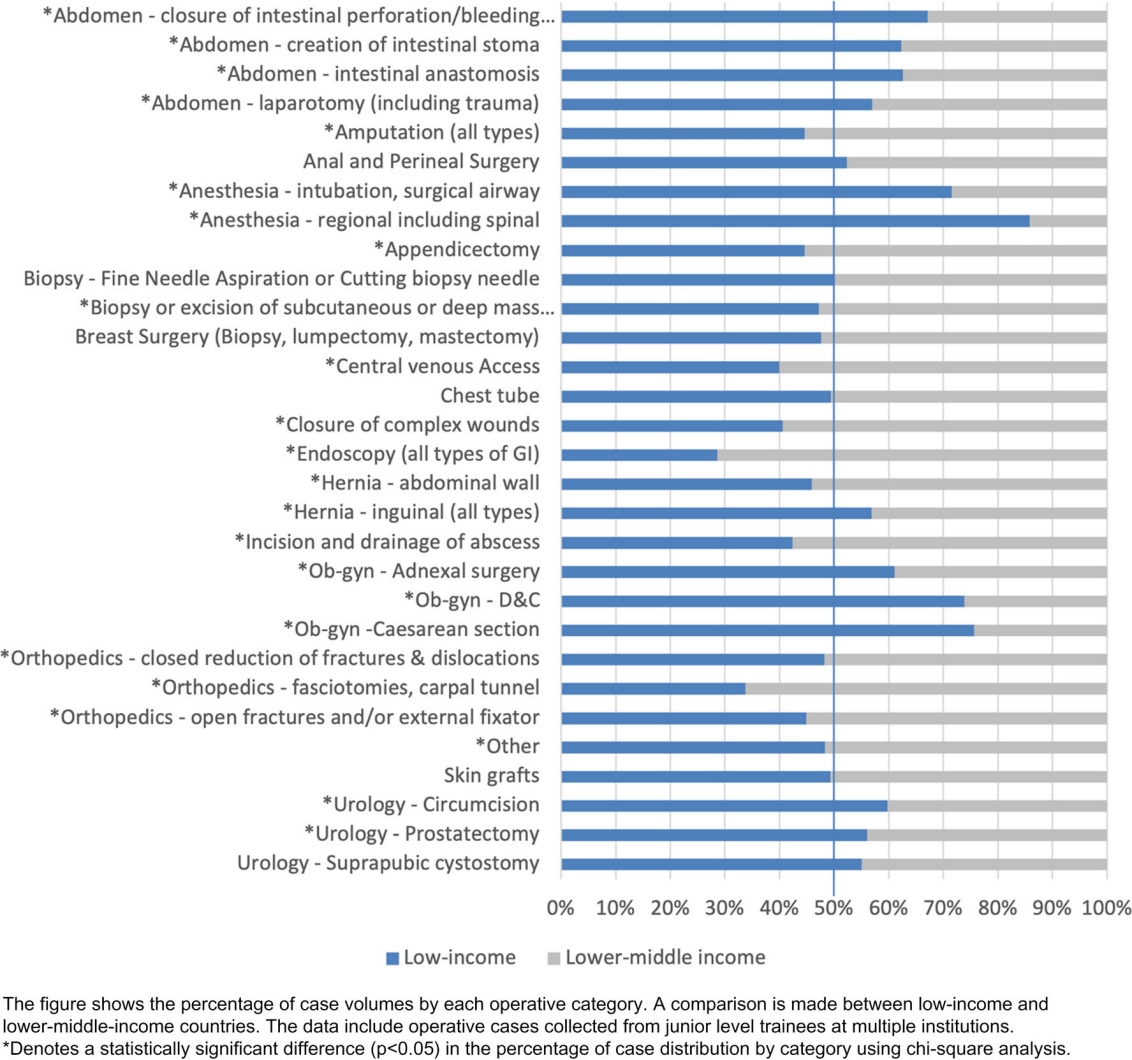
Percentage of case volumes by category for junior trainees compared between low-income and lower-middle-income countries

Figure 3 demonstrates operative volumes of various procedures across different countries for MCS trainees to better understand regional variation. Figure 4 shows a comparison between low- and lower-middle-income countries of the distribution of cases.

## Discussion

Surgical trainees in the ECSA region have a diverse operative experience. With all trainees obtaining the overall number of minimum case experience, there remains a gap between the guideline minimum number of cases by category and the observed experience of trainees. Most trainees were unable to meet the guidelines. In the FCS years, particular categories were difficult to achieve, including breast, gynecology, head & neck, thoracic surgery, urology, and vascular surgery. Similarly, in the MCS years, some procedures were not logged as frequently as recommended by the guidelines, including endoscopy, anesthesia, and urology. Essential surgeries of skin grafts, chest tube thoracostomy, and obstetrics & gynecology [20] also had less than 50% of trainees recording guideline minimums during their MCS years. These deficiencies highlight the experience of current trainees. They may help with either future revisions of guidelines and requirements of trainees or efforts to facilitate and ensure trainee exposure to the desired case minimums.

Operative minimums are considered necessary in the region [16], but the optimal target number identified by regional surgical educators would not be reached within this cohort of trainees. This finding could suggest that the guideline minimums may not be achievable and other objectives for surgical training should be prioritized, such as simulation [21]. The surgical learning curve for a particular procedure requires a wide range from 25 to 750 operations [22]. While higher case volumes have been positively correlated with patient outcomes [22–25], procedural numbers do not always correlate with trainee autonomy and learning [26]. The development of autonomy in the operating room is critical to creating competent, safe, and independent surgeons while not compromising patient safety [27, 28]. In the future, adopting entrustable professional activities for evaluation may limit the role and focus on specific case minimums [29, 30]. Regardless, some amount of operative experience remains necessary. This dataset represents surgical trainees' current realities and practices in the ECSA region.

We analyzed case volumes within various countries to understand better the diverse populations and diseases within the ECSA region. There are notable differences between countries, which could help decision-makers in each country to further refine minimum targets for their location. We also revealed trends of certain types of cases in countries with differing development indices. Lower middle-income countries recorded more cases with orthopedics, endoscopy, central venous access, and amputations. This is a similar finding from a study focusing on endoscopy conducted at multiple training institutions throughout Africa [31]. While there may be distinctive disease patterns to describe these findings, the changing dynamics of surgical education, specialization, or resource availability should also be considered. There may be an impact from the materials necessary for a given procedure or an increase in the burden of trauma with more motor vehicles. Lower-income countries had more experience with urology, anesthesia, and obstetrics & gynecology, which may reflect the availability, or lack thereof, of other care providers in those settings.

Our study has several limitations. As a retrospective analysis of case logs, it depends upon trainees' initiative to input their cases. There may be missing or inaccurate cases as there is no external validation. However, program directors familiar with the trainee’s experience must review a summary of the trainee’s case log before a trainee is allowed to undertake accrediting examinations, which provides some measure of accountability. Limitations exist on the classification of countries by development indices, but such comparisons should hopefully help policymakers and others interested in global surgery to understand the training environment. A substantial number of cases did not fall within the defined categories. These non-classified cases certainly add to the trainee's experience, even if they do not fulfill the guideline minimums for each category. Additionally, the COVID-19 pandemic impacted operative case volumes worldwide [32], and we did not assess the impact of this phenomenon. Further exploration of this impact and its long-term consequences is warranted in our setting.

Surgical trainees in East, Central, and Southern Africa have diverse operative training experience. Our findings should help surgeons, educators, and policymakers to understand better the current situation for surgical care and training in the region. Trainees fulfill the overall case volume requirements. If case minimums for categories are deemed appropriate, further exploration of how to meet the demands may be necessary. Innovations that ensure adequate operative experience, which contributes to training safe, competent, and independent surgeons, should continue to be explored within the ECSA region.
